# The influence of the age of dementia onset on college students’ stigmatic attributions towards a person with dementia

**DOI:** 10.1186/s12877-020-1505-4

**Published:** 2020-03-14

**Authors:** Perla Werner, Lilach Raviv-Turgeman, Patrick W. Corrigan

**Affiliations:** 1 Department of Community Mental Health, University of Haifa, Haifa, Israel; 2 Illinois Institute of Technology, Chicago, USA

**Keywords:** Attributional model, Dementia, Young-onset dementia, Late-onset dementia, Public stigma

## Abstract

**Background:**

Research in the area of public stigma and Alzheimer’s disease (AD) is limited to examining stigmatic beliefs towards persons aged 65 and over (i.e., persons with late-onset dementia). The aim of the present study was to compare college students’ stigmatic attributions towards an older and a younger person with AD, using an attributional model of stigma.

**Method:**

A cross-sectional study was conducted with 375 college students (mean age = 25.5, 58.9% female, 64.3% Jewish) who answered a computerized, self-administered, structured questionnaire after being presented with one of two randomly distributed vignettes varying in the age of the person with AD – 80 or 50 years of age. Cognitive, emotional and behavioral attributions of stigma were assessed using an adapted version of the Attribution Questionnaire. Other variables examined included background information, experiences and concerns about developing AD. T-tests and Ordinary Least Square (OLS) hierarchical regressions were used to analyze results.

**Results:**

Similar to previous studies, students’ levels of dementia stigma were low to moderate. Negative attributions were consistently and significantly higher (β = .17 to .33, *p* < .01), and positive attributions were significantly lower (β = −.26, p < .01) when the target person was younger rather than older.

**Conclusion:**

The differences in stigmatic beliefs towards a younger and older person with AD point to the theoretical and practical importance of clearly stating the age of the target person in stigma studies as well as in programs aimed at reducing public stigma towards persons with AD.

## Background

Driven by the increasing rates of dementia worldwide [1], public health and research attention is currently dominated by efforts to decrease stigmatic beliefs towards persons with dementia, in general, and Alzheimer’s disease (AD), in particular. This is reflected in all National Dementia Strategy programs implemented in a variety of countries [2], as well as in the increasing amount of studies assessing stigma among the general public (see reviews by Herrmann et al. 2018 [3]; Nguyen & Li, 2018 [4];Werner, 2014 [5]). Despite the importance of the studies assessing public stigma towards persons with AD, it should be noted that they have predominantly concentrated on stigma directed towards persons with late-onset dementia (LOD). While LOD, defined as onset dementia at the age of 65 and over, is the most common type of dementia, assessing stigmatic beliefs towards persons with onset dementia under the age of 65 – called young-onset dementia (YOD) – is very important for several reasons. First, although sharing the same neuropathological characteristics, both types of dementia differ in several features such as the rate of progression of the disease, genetic characteristic, and the presentation of behavioral problems [6]. Second, an extensive body of research has demonstrated that sigmatic experiences are common in the lives of persons with YOD, as well as among those surrounding them, such as family and professional caregivers [7–10]. Finally, since the internalization of stigma is closely associated to the stigmatic beliefs held by the public [11–13], evaluating public stigma towards younger persons with the disease – i.e., with young-onset dementia - is of the utmost importance.

Thus, the aim of the present study was to address this gap by comparing college students’ stigmatic beliefs towards an older and a younger person with AD, using an attributional model of stigma, which assumes that stigma includes three types of attributions: cognitive attributions or stereotypes about the person with the disease; emotional attributions, including negative and positive emotions; and behavioral attributions, including behavioral discrimination as well as willingness to help. Wile this conceptualization was developed originally for mental illness stigma [14], a recent study demonstrated that public stigma in the area of dementia is characterized by a similar cognitive, emotional, and behavioral process [15].

## Methods

### Study design and participants

This study used a cross-sectional design with a convenience sample of college students.

Using Green formula [16] the minimum sample required was 322. By assuming 30% dropout rate, a total of 494 college students were asked to participate in the study. Of these, 119 were excluded: nine because of language problems and 110 because they did not complete the questionnaire in its entirety. Thus, data from 375 students were available, rendering a response rate of 75.9%. The majority of the participants were female (58.9%), Jewish (64.3%), born in Israel (58.4%), not married (86.4%). Regarding their study characteristics, the majority were undergraduate students (85.6%), half (51.5%) studied social sciences, 31.5% exact sciences, and the rest humanities and health studies. Their mean age was 25.5 years (SD = 4.8, range 18–51).

### Measures

The following instruments were used:

#### Dependent variables - public stigma towards a person with AD

An adapted version of the Attribution Questionnaire 27 [AQ-27; 14] was used. The adaptation entailed replacing mental illness with AD, and the inclusion of two items assessing lack of aesthetics: To what extent do you think Sara is filthy/smelly?). The inclusion of items assessing aesthetic was based on previous findings stressing the importance of these cognitive attributions for dementia stigma [13, 15]. All items were rated on a 9-point, Likert-type scale, ranging from 1 = not at all to 9 = very much, and tapping three dimensions of stigma – cognitive attributions (dangerousness, responsibility, and lack of aesthetics), emotional reactions (negative and positive), and discriminatory behavior (segregation, treatment coercion, and helping behaviors). The adapted version of the instrument was validated in a previous study, and good to very good internal reliability (Cronbach alphas ranging from .70 for responsibility to .86 for negative emotions, were found [15].

#### Independent variables

These included socio-demographic characteristics, and health beliefs regarding AD**.**

***Socio-demographic characteristics*** included age, gender (male and female), majority (Jewish) or minority (non-Jewish) group, and area of study (health and other).

***Experiences and concerns of developing AD***: These included familiarity with the disease, and worry about developing it.

*Familiarity* was assessed by asking participants if they knew someone with Alzheimer’s disease among their relatives or acquaintances.

*Worry about developing Alzheimer’s disease* was assessed by a single question: “How much do you worry that you will develop Alzheimer’s disease?” Answers were rated on a 5-point Likert-type scale, ranging from 1 = not at all worried to 5 = very worried.

### Procedure

Participants were recruited opportunistically from various colleges in the Northern part of Israel. They were asked to answer a computerized structured questionnaire after being presented with a vignette developed and used in a previous study [17], but describing a female instead of male person with AD (Sara), aged 80 years old (LOD) or 50 years old (YOD). Besides of the age of the person, both vignettes were identical. Each version was assigned randomly, with 159 participants being exposed to the YOD vignette and 211 to the LOD vignette. No statistically significant differences were found in the socio-demographic or academic characteristics of the participants in each group or in their health beliefs regarding AD. It took approximately 15 min to complete the questionnaire.

### Statistical analyses

Descriptive statistics (percentages, means, and standard deviations) were used to describe the sample and the main variables. T-tests were used to assess differences in attributions of stigma according to the age of the person with AD described in the vignette. Finally, Ordinary Least Squares (OLS) hierarchical regressions were conducted in order to examine the effect of the age of the person with AD on stigmatic attributions in each one of the stigma dimensions. In the first step of the regressions, we included the age of the person with AD as presented in the vignettes. In the second step, sociodemographic factors were entered, followed by health beliefs about Alzheimer’s disease, in the third step. We tested for multicollinearity, and the results indicated that it was not a concern in our model. Variance inflation factor (VIF) did not exceed 2.3.

### Ethical considerations

The study’s protocol was approved by the Ethics Committee of the University of Haifa. All potential participants were given and read an informed consent form describing the importance of the study, its anonymity, and the possibility to refuse to participate without any consequences. The students were required to sign an informed consent form before starting to answer the questionnaire.

## Results

**Fig. 1:**
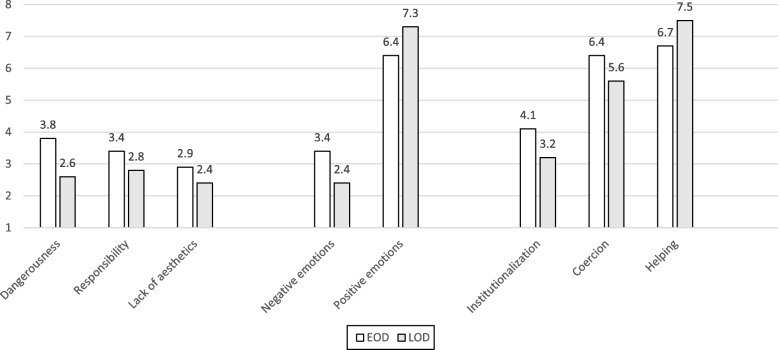
College students’ stigmatic attributions regarding persons with YOD and LOD

Overall, as can be observed in Fig. 1, regardless of which vignette was presented and with the exception of coercion, college students’ stigmatic attributions were below the neutral score of 4.5, reflecting low to moderate levels of stigma towards a person with AD. However, participants exposed to the vignette presenting a person with YOD reported significantly higher levels of cognitive attributions, negative emotions, and attributions of segregation and coercion, than those exposed to the LOD vignette. Positive emotions and willingness to help were significantly lower in the group exposed to the YOD, compared to those exposed to the LOD vignette. Moreover, results of the hierarchical regressions (Table 1) showed that the age of the person with AD had the strongest influence on explaining dangerousness (R^2^ = .10), negative reactions (R^2^ = .10), positive reactions (R^2^ = .06), treatment coercion (R^2^ = .04), and helping behaviors (R^2^ = .06). While age, gender, and area of study were not significantly associated with any of the stigma variables, participants pertaining to a minority (non-Jewish) group reported higher levels of stigmatic beliefs compared to those pertaining to a majority (Jewish) group. More specifically, this variable was the most important determinant of lack of aesthetics (it increased the explained variance by 4% when added in Step 2), as well as of responsibility (it increased the explained variance by 10% when added in Step 2), and institutionalization (it increased the explained variance by 7% when added in Step 2). Experiences and concerns about developing AD had a very small influence on explaining stigma attributions for all dimensions.

**Table 1:** Hierarchical regressions assessing the effect of the age of the person described in the vignette (*n* = 375)

	Cognitive dimension			Emotional reactions		Behavioral attributions		
	Dangerousness	Lack of Aesthetics	Responsibility	Negative Reactions	Positive Reactions	Institutionalization	Treatment Coercion	Helping
***Step 1***								
LOD/YOD	.33^***^	.17^**^	.20^**^	.33^***^	−.26^***^	.21^***^	.19^***^	−.26^***^
**Adjusted R**^**2**^	.10	.02	.03	.10	.06	.04	.04	.06
***Step 2***								
Age	.01	.05	.03	.01	.03	−.07	.03	−.03
Gender	−.01	.03	−.05	−.03	.10	−.07	.01	.10
Jewish/Non-Jewish	.21^***^	.19^*^	.29^***^	.20^**^	.01	.27^***^	.18^**^	−.02
Area of study	−.09	−.07	−.17^**^	−.13^*^	.10	.02	.09	.05
**Adjusted R**^**2**^	.14	.06	.13	.15	.06	.11	.06	.06
***Step 3***								
Familiarity with AD	.02	−.14^*^	.11^*^	−.01	−.07	.02	−.17^**^	−.01
Concern about AD	.04	.04	.02	.06	.09	.10	.20^***^	.14^*^
**Total Adjusted R**^**2**^	.14	.07	.15	.15	.07	.12	.10	.08

^*^p < .05, ^**^p < .01, ^***^p < .00

## Discussion

This study investigated whether differences exist among college students’ stigmatic attributions towards a person with YOD or LOD. Overall, similar to other studies conducted with the general public [15, 18–23], stigmatic attributions towards a person with AD were low to moderate in our sample of relatively young college students. This calls for special attention, particularly since it was established that stigmatic processes, in general, begin at a young age [24], and specifically in regards to dementia, stigmatic attributions increase with age [22, 25, 26] .

Most importantly, our study expands these findings by demonstrating that stigmatic attributions elicited by a person with AD vary according to the age of the target person. Indeed, college students consistently reported higher stigmatic attributions when presented with a younger person with dementia than with an older one. Several explanations might be provided for this finding: some relate to the concept of stigma in the area of AD and some to general attitudes towards older persons.

*Public stigma* is defined as laypersons’ perceptions of a person or a group as having a cue or mark which makes them different or abnormal [27]. Given the characteristics of AD and its clear association with older age [28], we can hypothesize that the higher levels of stigmatic beliefs reported toward the younger person with AD were a result of her being perceived as “different” or “abnormal”. This explanation is supported by studies examining persons with YOD and their caregivers, which described stigma as a reoccurring theme because of the uncommon situation of encountering a relatively young person with the disease [29]. Moreover, it has been stated that laypersons’ stigmatic beliefs are associated with perceptions of threat associated with the stigmatized group [30]. In view of the relatively young age of our sample, we can assume that the feelings of threat will be higher for those exposed to the vignette describing a person closer to their age.

Alternatively, we can assume that the differences in stigmatic beliefs associated with the age of the person with AD do not stem, as suggested above, from higher stigmatic beliefs directed towards the younger person, but from lower stigmatic beliefs towards the older person with AD caused by paternalistic stereotypes. While *paternalistic stereotypes*, defined as attributions attached to a person as needing care and help, were previously studied mainly in regard to persons with mental illness [31], studies showed that older healthy targets also elicit more positive emotions than younger targets, even if they are perceived as less competent [32, 33].

Finally, the age of the person described in the vignette remained a significant predictor of all stigmatic attributions, even after including other variables. Moreover, it emerged as the most important determinant for two negative attributions (dangerousness, and negative reactions), and the second most important determinant for the rest of the attributions, apart from ethnicity. These findings call for future studies in the area of dementia stigma to explicitly state the age of the person with the disease.

In addition to the contribution of the age of the person with AD to the explanation of stigmatic attributions, our findings showed that pertaining to a minority (non-Jewish) group was consistently associated with increased stigma. A similar trend was found in an Israeli sample of high school students [34], stressing the importance of cultural factors to the formation of stigmatic beliefs. Finally, the lack of significant relationships with health beliefs is noteworthy, as this is a central factor in attribution models of stigma [14]. While this might be a result of the relatively young age of our sample, future studies should try to further examine these associations.

Several limitations should be noted before we discuss the implications of our results. First, the study employed a non-random sample. Participation in the study was voluntary and no information was available about the characteristics of those who did not participate. Finally, the cross-sectional design of our study does not allow for a causal interpretation of the results.

Our findings should also be considered in the context of our measurement methods. The use of self-reported measures might be associated with social desirability bias and item non-response. However, only 11 to 14% of the participants did not answer one of the attribution items. Moreover, we hope that the anonymity of the questionnaires encouraged students to be honest in their responses. Another limitation might be the use of vignettes, which do not necessarily reflect real-life situations [35]. However, vignettes are commonly used to assess stigmatic beliefs in a variety of conditions [36, 37], and they have been especially useful for testing hypotheses [17, 20, 38–40]. Finally, it should be noted that our vignettes did not use the terms “young/late onset dementia”, but rather varied the age of the person described in them. Future studies might want to assess the effect of using these labels on stigmatic beliefs towards a person with AD.

## Conclusions

Notwithstanding these limitations, this study has important implications. First, it supports recent findings [10] showing that the use of an expanded model of Attribution Theory provides an adequate conceptual framework for understanding dementia stigma. Second, the differences found in the stigmatic beliefs attributed to a younger or an older person with AD point to the theoretical and practical importance of clearly stating the age of the target person in stigma studies and in anti-stigma campaigns in the area of AD.

## Data Availability

The datasets used are available from the corresponding author on reasonable request.

## Abbreviations

AD: Alzheimer’s disease
LOD: Late-onset dementia
OLS: Ordinary Least Squares
VIF: Variance inflation factor
YOD: Young-onset dementia
